# Patient satisfaction and perceived quality of care: evidence from a cross-sectional national exit survey of HIV and non-HIV service users in Zambia

**DOI:** 10.1136/bmjopen-2015-009700

**Published:** 2015-12-30

**Authors:** Emily Dansereau, Felix Masiye, Emmanuela Gakidou, Samuel H Masters, Roy Burstein, Santosh Kumar

**Affiliations:** 1 Department of Public Health, Institute for Health Metrics and Evaluation, Seattle, Washington, USA; 2 Department of Economics, University of Zambia, Lusaka, Zambia; 3 Department of Public Health, University of North Carolina, Chapel Hill, North Carolina, USA; 4 Department of Economics & International Business, Sam Houston State University, Huntsville, Texas, USA

**Keywords:** Patient satisfaction, Perceived quality of care, Exit interview, HIV, Zambia

## Abstract

**Objective:**

To examine the associations between perceived quality of care and patient satisfaction among HIV and non-HIV patients in Zambia.

**Setting:**

Patient exit survey conducted at 104 primary, secondary and tertiary health clinics across 16 Zambian districts.

**Participants:**

2789 exiting patients.

**Primary independent variables:**

Five dimensions of perceived quality of care (health personnel practice and conduct, adequacy of resources and services, healthcare delivery, accessibility of care, and cost of care).

**Secondary independent variables:**

Respondent, visit-related, and facility characteristics.

**Primary outcome measure:**

Patient satisfaction measured on a 1–10 scale.

**Methods:**

Indices of perceived quality of care were modelled using principal component analysis. Statistical associations between perceived quality of care and patient satisfaction were examined using random-effect ordered logistic regression models, adjusting for demographic, socioeconomic, visit and facility characteristics.

**Results:**

Average satisfaction was 6.9 on a 10-point scale for non-HIV services and 7.3 for HIV services. Favourable perceptions of health personnel conduct were associated with higher odds of overall satisfaction for non-HIV (OR=3.53, 95% CI 2.34 to 5.33) and HIV (OR=11.00, 95% CI 3.97 to 30.51) visits. Better perceptions of resources and services were also associated with higher odds of satisfaction for both non-HIV (OR=1.66, 95% CI 1.08 to 2.55) and HIV (OR=4.68, 95% CI 1.81 to 12.10) visits. Two additional dimensions of perceived quality of care—healthcare delivery and accessibility of care—were positively associated with higher satisfaction for non-HIV patients. The odds of overall satisfaction were lower in rural facilities for non-HIV patients (OR 0.69; 95% CI 0.48 to 0.99) and HIV patients (OR=0.26, 95% CI 0.16 to 0.41). For non-HIV patients, the odds of satisfaction were greater in hospitals compared with health centres/posts (OR 1.78; 95% CI 1.27 to 2.48) and lower at publicly-managed facilities (OR=0.41, 95% CI=0.27 to 0.64).

**Conclusions:**

Perceived quality of care is an important driver of patient satisfaction with health service delivery in Zambia.

**Strengths and limitations of this study**

- To the best of our knowledge, this study is the first to examine the association between perceived quality of care and patient satisfaction through exit interviews across Zambia.
- Adequacy of medical resources and provider conduct and practices were significant predictors of overall patient satisfaction.
- Facility characteristics such as management type, location and level of facility were important determinants of patient satisfaction.
- Methodological concerns associated with over-representation of users and lack of causality are acknowledged.

## Background

For nearly 25 years, the World Health Organization (WHO) has identified meeting individuals’ universally legitimate expectations as a key health system objective.^1^ Patient satisfaction and ratings have been given increasing importance for measuring the quality of health services and are routinely used in developed countries for continuous quality improvement and value-based incentive payments.^2^ ^3^ In addition to the intrinsic importance of meeting reasonable expectations, patient satisfaction and perceptions are associated with healthcare utilisation and choice of provider.^4–6^ Studies have also linked satisfaction to treatment adherence for HIV patients, which has important implications for individual patient outcomes and preventing resistance to antiretroviral drugs (ARVs).^7^ ^8^

This study focuses on patient satisfaction and perceptions in Zambia, a sub-Saharan African country with 16.2 million citizens.^9^ Approximately 80% of Zambian health facilities are publicly managed, and the government has worked to decentralise decision-making to the district level since 1991.^10^ While service utilisation has improved in recent years, it continues to be a major concern; in the 2013–2014 Demographic and Health Survey (DHS), 64% of deliveries were performed by a skilled provider and 66% of children received medical attention for diarrhoea.^11^

HIV is a priority issue in Zambia, where adult prevalence was estimated at 13.3% in the 2013–2014 DHS.^11^ In 2012, Zambia dispensed ARVs to over 500 000 patients at 564 facilities, most of which were stand-alone vertical facilities associated with a general clinic.^12^ ^13^ Currently, the National Aids Strategic Framework emphasises moving towards a model that integrates HIV prevention, diagnosis and treatment with other primary health services.^14^ While integration is still underway, a scaled-up pilot at 12 primary care clinics in Lusaka found that integration offered management and organisational advantages, but not human resource or infrastructure gains.^15–17^

Despite evidence that satisfaction can drive the utilisation of antiretroviral therapy (ART) and other priority services, Zambia lacks a systematic means of monitoring and responding to patient opinions. Zambian patient perceptions have only been measured in a handful of small studies in limited populations. A study of maternity care in Lusaka found that while 89% of women rated care as ‘good’ or ‘very good’, 21% were shouted at, scolded or otherwise treated badly during delivery.^18^ In another survey, the majority of patients were not satisfied with the quality of care for sexually transmitted diseases at an urban health centre.^19^ A third study was conducted across three districts and found average district-level adult satisfaction scores ranging from 70% to 76%.^20^ Adults gave lower satisfaction ratings in periurban areas in this study, suggesting that satisfaction varies by facility type and location.^20^ No prior national studies have described the extent of this variation or examined factors that explain it.

Research in other developing settings have identified a variety of factors that may drive satisfaction, including provider attitudes and respectfulness, technical provider ability, wait time, drug availability, facility appearance, and patient expectations.^21–25^ The findings have varied depending on the country and setting, leaving a gap in knowledge as to what drives satisfaction in the Zambian context.

In this study, we report findings from exit surveys of patients receiving HIV and non-HIV services at a diverse sample of facilities across Zambia. We describe levels and variations in patients’ overall satisfaction, as well as their perceptions of specific interpersonal and technical aspects of care. Additionally, we examine how individual characteristics, facility-level factors, and perceptions of specific aspects of care relate to overall satisfaction, to highlight areas for potential interventions to improve patient satisfaction in Zambia.

## Methods

### Sample and data collection

The exit interviews were conducted between December 2011 and May 2012 across 16 Zambian districts as part of the Access, Bottlenecks, Costs, and Equity (ABCE) project. The details of this project are documented elsewhere and available online.^26^

A two-step stratified random sampling process was used to select health facilities. First, Zambia's districts (72 at the time, currently 103) were stratified on the basis of average household wealth, population density and skilled birth attendance (SBA) coverage. One district was randomly selected from each wealth–population–SBA category, in addition to the capital, Lusaka. In each selected district, we selected all hospitals, two urban health centres, three rural health centres, and a quota of associated health posts. The exit interviews were conducted at a subset of the facilities selected for the overall ABCE project. Our study reports on interviews conducted at 104 facilities. Compared with all facilities in Zambia, we oversampled hospitals and urban health centres and undersampled rural health centres and health posts to allow for platform-specific analyses (see online supplementary appendix table 1). Our sample is representative of the Zambian population and health delivery system, except that we oversampled hospitals to allow for separate analyses of hospital data. The sample of patients who sought care was also skewed towards females, which is expected due to several factors including women seeking maternal health services and a higher HIV prevalence among women (15.1%) than men (11.3%).^11^

At each facility participating in the exit survey, 30 patients were systematically sampled as they exited. Sampling intervals varied from every patient to every four patients, depending on the patient volume reported by the facility manager. The sample size of 30 patients at each facility was estimated using the Kish method with the following assumptions: patient satisfaction rate of 10%, precision of 5%, α of 1%, design effect of two, and non-response rate of 20%. The estimated sample from the Kish method was further adjusted to allow for robust subgroup analyses (eg, HIV vs non-HIV; hospital vs health clinic; rural vs urban). Interviews were conducted over at least two days at each facility. Patients were required to be 15 years or older and in an appropriate physical and mental state to be eligible to complete the survey. If a patient was too young or otherwise ineligible, an eligible attendant was asked to answer on their behalf when possible. Verbal consent was obtained from all respondents, and surveys were conducted in a location where the facility staff and other patients were not present.

Trained research assistants recorded exit interview responses electronically using the DatStat data collection software. On a daily basis, data were uploaded to a database accessible from Seattle, where they were continually verified for quality during the collection process. The median interview time was nine minutes.

### Facility survey instrument

At each health facility, research assistants interviewed facility administrators to collect information about facility resources, staffing, management and practices. Facility level and management were verified against a facility roster provided by the Ministry of Health (MOH).

### Exit survey instrument

The exit instrument drew questions from established patient exit and household surveys, which in-country partners tested and modified to fit the Zambian context. Demographic questions were based on the Zambian DHS.^27^ Questions about visit circumstances and costs were adapted from the World Health Survey.^28^

We measured patients’ overall satisfaction with the facility with the following question from the Consumer Assessment of Healthcare Providers and Systems Adult Visit questionnaire: *Using any number from 1 to 10, where 1 is the worst facility possible and 10 is the best facility possible, what number would you use to rate this facility?*^29^ ^30^

The survey also captured how patients perceived the quality of specific aspects of the facility and its providers, based on a validated questionnaire developed by Baltussen *et al*^31^ that has been used in other developing settings.^32^ ^33^ Patients were asked to rate 25 aspects of the facility on a five-point Likert scale: very bad, bad, moderate, good or very good. The majority of questions were answered by over 95% of patients, but we excluded five questions to which over 10% of patients responded ‘not applicable’, ‘don't know’, or ‘decline to respond’. These five questions concerned: adequacy of doctors for women, ease of making payment arrangements, time doctors allow for patients, availability of good doctors, and provider's follow-up with patients.

### Condensing perceived quality responses

We then used principal component analysis (PCA) with orthogonal rotation to examine the structure of the remaining 20 perceived quality questions (see online supplementary appendix table 2). The analysis identified five components with eigenvalues ranging from 0.94 to 7.8, which explained 62% of the variance. The factors aligned with theoretical domains and can be interpreted as: (1) health personnel practices and conduct, (2) adequacy of resources and services, (3) healthcare delivery, (4) accessibility of care, and (5) cost of care. The specific questions under each domain are listed in online supplementary appendix table 2. The factor with an eigenvalue under 1 (accessibility of care) was retained because the variables it contained were theoretically grouped and not otherwise represented. Cronbach's α coefficients for each grouping ranged from 0.70 to 0.90, which met the generally accepted threshold of 0.70 and was comparable to or better than studies conducting similar exercises.^34^

To condense the information for each domain, we created a new variable that was the per cent of questions within the domain which the respondent rated ‘good’ or ‘very good’. We opted to examine the responses in this categorical manner rather than as continuous averages because (1) Likert scales from very bad to very good are not truly continuous and (2) research shows that patients typically rate facilities favourably, and therefore the important distinction is achieving the very highest ratings.^35–37^ If a patient did not answer a given question, we took the per cent among the questions that were answered.

### Ordered logistic regression analysis

We used random-effects ordered logistic regression models to examine how overall satisfaction (rated from 1 to 10) was related to objective patient, facility and visit factors, as well as patient perceptions of specific aspects of care (measured with the 5-point Likert scale).

The unit of analysis was the patient, and the outcome for all models was the patient's overall rating of the facility out of 10 (described above in *measuring satisfaction*). An ordered model was selected because the outcome scale was ordered but not truly continuous. Additionally, since the outcome variable was skewed towards higher ratings, we grouped all responses below six into a single category for the purpose of the regression models (see online supplementary appendix figure 1).

The first model examined how facility, patient and visit characteristics were associated with overall satisfaction. Independent variables were selected *a priori* based on relationships previously identified in the literature. Facility variables included facility type (hospital or health centre/post), location (urban or rural), and management (public or non-governmental organisation [NGO]/private). Demographic variables included age, self-rated overall health, ethnicity, sex, education level, and a binary indicator of whether the respondent was the patient or an attendant. Variables surrounding visit circumstances included travel time, wait time, and type of provider seen. We did not include whether or not the patient paid a user fee as this was largely determined by facility management—public and NGO facilities typically offer free services while private facilities often charge fees.

The second model looked at how patients’ perceptions of particular domains of care related to their overall perception, to identify which aspects are most influential. The predictor variables in this case were the five summary perceived quality variables (described above in *condensing perceived quality responses*): health personnel practices and conduct, adequacy of resources and services, healthcare delivery, accessibility of care, and cost of care.

Our final combined model included all of the facility, patient, visit, and perceived quality predictors from the first and second models. This allowed us to examine whether any facility, patient or visit characteristics were associated with overall satisfaction independent of how the patient rated specific aspects of care.

All models included facility random effects to account for unmeasured facility characteristics, and we estimated robust standard errors (SEs) to account for intragroup correlation within facilities. Patients missing one or more covariates were excluded from all regression analyses.

Our sample contained a substantial number of patients receiving HIV-related services; we analysed these patients separately from those receiving other services because HIV care may involve specialised staff, equipment and drugs, and because HIV often receives unique policy attention based on the large burden it poses in Zambia.

We additionally conducted sensitivity analyses to test for effect modification by facility management, facility location, facility level and respondent identity (patient or attendant). To do this, we conducted the same analyses described above, stratified by the characteristic of interest, rather than by the HIV visit or not.

Data management and analysis were conducted in Stata V.13.1.

## Results

The response rate among eligible patients was 97% and a total of 2789 exit interviews were conducted. After excluding 61 patients who lacked an associated facility survey, 31 who had not received services that day and 76 who did not provide an overall satisfaction score, our sample included 2528 patients from 104 facilities. Of these, 413 had received HIV-related services that day and 2115 had received other services. An additional 203 patients were missing information for one or more variables in the regression and were excluded from all regression analyses.

Seventy-one per cent of survey respondents were the patients themselves, while 29% were attendants. Of the attendant respondents, 94% were accompanying a patient under the age of 15 years.

### Facility characteristics

The sample of 104 facilities included 14 urban hospitals, 7 rural hospitals, 51 rural health centres/posts, and 32 urban health centres/posts.

The majority of exit interviews were conducted in health centres or posts (80%) and in facilities managed by the government (81%) (table 1). Overall, around half of the visits occurred in urban areas, though HIV visits more often occurred at urban facilities (59%), compared with other types of care (45%).

**Table 1:** Characteristics of sampled patients

	Non-HIV	HIV	All		
	n=2115 (%)	n=413 (%)	n=2528 (%)	p Value*	Missing (%)
*Patient demographics*					
Respondent					
Patient	1412 (67)	389 (94)	1801 (71)	<0.01	0 (0)
Attendant	703 (33)	24 (6)	727 (29)		
Age (median, IQR)	23 (6–33)	32 (25–41)	25 (11–35)	<0.01	
0–5	486 (23)	5 (1)	491 (19)	0	0 (0)
6–17	278 (13)	18 (4)	296 (12)		
18–39	999 (47)	272 (66)	1271 (50)		
≥40	352 (17)	118 (29)	470 (19)		
Self-rated health					
Poor	242 (11)	39 (9)	281 (11)	0.56	3 (<1)
Fair	565 (27)	122 (30)	687 (27)		
Good	693 (33)	126 (31)	819 (32)		
Very good	422 (20)	86 (21)	508 (20)		
Excellent	191 (9)	39 (9)	230 (9)		
Ethnicity					
Tonga	455 (22)	103 (25)	558 (22)	0.04	3 (<1)
Chewa Nyanja	479 (23)	76 (18)	555 (22)		
Bemba	309 (15)	74 (18)	383 (15)		
Baroste Lozi	206 (10)	31 (8)	237 (9)		
Nsenga	151 (7)	38 (9)	189 (7)		
Other	513 (24)	90 (22)	603 (24)		
Education (respondent)					
Pre-primary/none	199 (10)	28 (7)	227 (9)	0.19	29 (1)
Primary	794 (38)	156 (38)	950 (38)		
Post-primary	1095 (52)	227 (55)	1322 (53)		
Sex (respondent)					
Female	1460 (69)	246 (60)	1706 (68)	<0.01	1 (<1)
Male	654 (31)	167 (40)	821 (32)		
*Facility characteristics*					
Facility type					
Hospital	358 (17)	159 (38)	517 (20)	<0.01	0 (0)
Health centre/post	1757 (83)	254 (62)	2011 (80)		
Location					
Urban/peri-urban	947 (45)	244 (59)	1191 (47)	<0.01	0 (0)
Rural	1168 (55)	169 (41)	1337 (53)		
Management					
Government	1748 (83)	296 (72)	2044 (81)	<0.01	0 (0)
NGO	174 (8)	98 (24)	272 (11)		
Private	193 (9)	19 (5)	212 (8)		
*Visit characteristics*					
Travel time in minutes (median, IQR)	30 (20–60)	45 (20–72)	30 (20–60)	0.57	83 (3)
Wait time in minutes (median, IQR)	45 (20–87)	60 (40–147)	50 (20–120)	0.04	55 (2)
Visit history					
First time at facility	1918 (91)	393 (95)	2311 (92)	0.01	6 (<1)
Visited facility previously	189 (9)	20 (5)	209 (8)		
Medical fees					
No fees paid	1784 (87)	395 (96)	2179 (89)	<0.01	70 (3)
Paid fee	264 (13)	15 (4)	279 (11)		
Main provider seen					
Doctor/clinical officer	596 (28)	152 (37)	748 (30)	<0.01	7 (<1)
Nurse	1152 (55)	244 (59)	1396 (55)		
Other	362 (17)	15 (4)	377 (15)		

*p Value comparing HIV and non-HIV patients using two-sided Student's t test for binary and continuous variables and χ^2^ test for categorical variables (H_0_:p_non-HIV_=p_HIV_).

NGO, non-governmental organisation.

### Patient characteristics

The majority of respondents were female (68%) and had a post-primary education (53%). Patients most often rated their overall health as ‘good’ (32%), while 9% rated it ‘excellent’ and 11% ‘poor’. The most common ethnicities were Tonga (22%) and Chewa Nyanja (22%). Only 1% of patients receiving HIV services were aged under 5 years, while 23% of those receiving other types of care were in the under-5 age group.

### Visit characteristics

A typical patient spent longer waiting at the facility (median of 50 min) than travelling to it (median of 30 min). Patients receiving HIV services waited and travelled longer than other patients: 38% of patients receiving HIV services waited over two hours compared with 23% of other patients. Only 4% of HIV-related visits involved any medical fees at the facility, compared with 13% of other visits. Among patients receiving non-HIV services, those visiting government-managed facilities paid fees less frequently (10%) than those at private (30%) and NGO facilities (23%).

### Patient satisfaction

**Figure 1:**
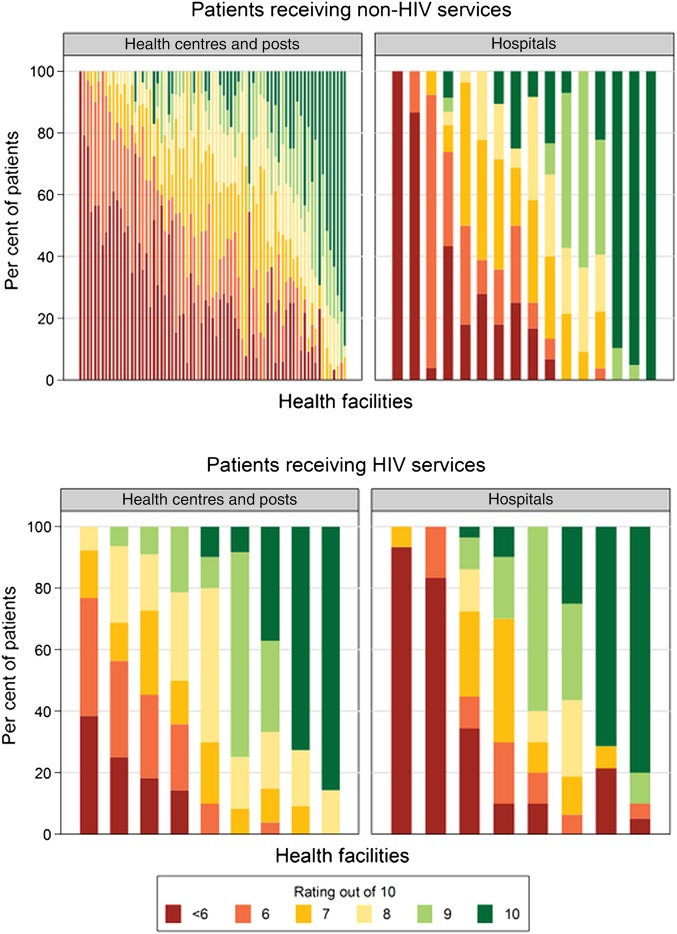
Composition of overall ratings, at facilities with at least 10 surveys conducted with patients of the specified visit type. Note: each vertical bar represents a health facility. Within each bar, each colour shows the proportion of patients interviewed at that facility that gave the rating associated with that colour in the legend.

Figure 1 shows the distribution of patients’ overall satisfaction scores at each facility. The average score on a 10-point scale was significantly higher for patients receiving HIV services (7.3) than others (6.9) (one-sided p<0.01). In total, 21% of patients receiving HIV services and 28% of other patients gave a satisfaction score of 5 or lower. The average satisfaction score at a given facility ranged from 3.2 to 10 for patients receiving non-HIV services, and from 3.7 to 9.7 for those receiving HIV services. Descriptively, satisfaction was higher at facilities that were urban (7.4) or privately/NGO managed (7.5) than those that were rural (6.6) or publicly managed (6.8).

### Perceived quality

Table 2 summarises patients’ perceptions of the 20 specific aspects of care, grouped into five domains. The best perceived aspect was cost of care (96% *very good* or *excellent*). All of the questions comprising the health personnel domain were rated *very good* or *excellent* by at least three-quarters of patients, with the exception of involvement in decision-making (64%). The lowest-rated domains were adequacy of resources and services, and accessibility of care. Less than half of all patients rated medical equipment adequacy (44%), facility spaciousness (46%), and wait time (46%) as *very good* or *excellent*. Medical equipment adequacy, drug availability, drug quality, treatment effectiveness, and cost of care were rated as *very good* or *excellent* significantly more often for HIV visits compared with other types of visits. The opposite was true of wait time and facility cleanliness.

**Table 2:** Perceived quality of specific aspects of care

	% Very good or excellent				
Dimension of perceived quality	Non-HIV care	HIV care	All patients	Difference between HIV and non-HIV (% points)	Two-sided p value (H_0_: p_non-HIV_=p_HIV_)
Health personnel practices and conduct					
Compassion and support	81	83	81	2	0.36
Respect shown	84	85	84	1	0.40
Reception by provider	82	81	82	1	0.77
Honesty of provider	85	85	85	0	0.95
Clinical examination quality	80	81	80	1	0.48
Privacy during examination	85	86	85	1	0.45
Patient involved in decision-making	65	62	64	-3	0.39
Adequacy of resources and services					
Medical equipment adequacy	42	54	44	12	<0.01
Facility cleanliness	57	52	56	-5	0.05
Waiting and examination room spaciousness	45	50	46	5	0.10
Drug availability	57	68	59	11	<0.01
Healthcare delivery					
Good diagnosis	79	83	79	4	0.38
Prescription of good drugs	80	84	81	4	0.07
Drug quality	82	87	83	5	0.01
Treatment effectiveness	82	87	83	5	0.01
Accessibility of care					
Hours of operation	67	70	67	3	0.25
Ease of obtaining drugs	75	78	75	3	0.15
Distance to health facility	55	50	54	-5	0.09
Waiting time	47	38	46	-9	<0.01
Cost of care					
Cost of medical care	96	99	96	3	<0.01

### Regression results

In the first models including demographic, facility and visit characteristics, rural location was associated with lower odds of overall satisfaction for patients receiving HIV services (odds ratio (OR)=0.25, 95% confidence interval (CI) 0.14 to 0.46) and non-HIV services (OR=0.16, 95% CI 0.11 to 0.22) (table 3). Patients receiving non-HIV care also had lower odds of high satisfaction at public facilities (OR=0.43, 95% CI 0.32 to 0.58), and higher odds at hospitals (OR=1.91, 95% CI 1.37 to 2.65). The only demographic factor significantly associated with overall satisfaction in these models was Tonga ethnicity for patients receiving HIV services (OR=0.50, 95% CI 0.28 to 0.88).

**Table 3:** Ordered logistic regression results examining demographic, facility, and visit factors as predictors of overall patient satisfaction

	Non-HIV services		HIV services		All patients	
	OR	(95% CI)	OR	(95% CI)	OR	(95% CI)
*Facility characteristics*						
MOH managed (ref: private/NGO)	0.43***	(0.32 to 0.58)	1.10	(0.68 to 1.77)	0.70*	(0.53 to 0.93)
Rural (ref: urban)	0.16***	(0.11 to 0.22)	0.25***	(0.14 to 0.46)	0.56***	(0.42 to 0.76)
Hospital (ref: health centre/post)	1.91***	(1.37 to 2.65)	1.25	(0.69 to 2.25)	3.02***	(2.28 to 4.00)
*Patient characteristics*						
Age	1.00	(0.99 to 1.01)	1.00	(0.97 to 1.02)	1.00	(1.00 to 1.01)
Male respondent	1.04	(0.86 to 1.26)	1.03	(0.55 to 1.92)	1.07	(0.90 to 1.27)
Education of respondent (ref: none)						
Primary	0.90	(0.67 to 1.20)	1.03	(0.33 to 3.24)	0.81	(0.60 to 1.11)
Post-primary	0.84	(0.61 to 1.16)	0.86	(0.26 to 2.84)	0.68*	(0.49 to 0.94)
Self-rated very good or excellent health	1.19	(0.94 to 1.50)	1.67	(0.86 to 3.23)	1.34*	(1.06 to 1.68)
Ethnicity (ref: other)						
Tonga	0.87	(0.69 to 1.10)	0.50*	(0.28 to 0.88)	0.98	(0.80 to 1.21)
Chewa Nyanja	0.84	(0.60 to 1.18)	0.90	(0.53 to 1.52)	0.87	(0.66 to 1.13)
Patient respondent (ref: attendant)	0.88	(0.68 to 1.13)	1.73	(0.81 to 3.70)	0.87	(0.70 to 1.08)
*Visit characteristics*						
Travelled an hour or more	0.83	(0.67 to 1.02)	0.81	(0.47 to 1.38)	0.88	(0.73 to 1.05)
Waited an hour or more	0.96	(0.78 to 1.18)	0.76	(0.47 to 1.20)	0.78**	(0.64 to 0.94)
Saw doctor or clinical officer (CO) (ref: nurse/other)	0.77	(0.58 to 1.04)	1.95	(0.87 to 4.35)	0.82	(0.63 to 1.06)
N	1927		398		2325	

The exponentiated coefficients shown here can be interpreted as the OR of giving a higher overall rating, for a one-unit increase in the predictor variable.

*p<0.05; **p<0.01; ***p<0.001.

MOH, Ministry of Health, Zambian government; NGO, non-governmental organisation.

There was evidence of effect modification by facility type in the sensitivity analysis (see online supplementary appendix table 3b–d). Among patients visiting hospitals, odds of satisfaction were significantly higher when the facility was publicly managed (OR=2.42, 95% CI 1.09 to 5.37); in contrast, at non-hospitals, odds of satisfaction were significantly lower when the facility was publicly managed (OR=0.24, 95% CI 0.13 to 0.42). Among patients at urban facilities, odds of satisfaction were higher if it was a hospital (OR=16.84, 95% CI 10.70 to 26.51), while at rural facilities, odds of satisfaction were lower if it was a hospital (OR=0.18, 95% CI 0.10 to 0.34).

In table 4, we explore how perceptions of specific aspects of care relate to overall satisfaction. Perceptions of health personnel practices and conduct had the strongest association with overall satisfaction for non-HIV (OR=3.05, 95% CI 1.95 to 4.77) and HIV (OR=9.49, 95% CI 2.51 to 35.93) patients. This was the only significantly associated domain for HIV patients, while for non-HIV patients adequacy of resources and services (OR=2.17, 95% CI 1.30 to 3.62) and accessibility of care (OR=2.52, 95% CI 1.69 to 3.79) were also significant predictors.

**Table 4:** Ordered logistic regression results examining perceived quality of domains of care as predictors of overall patient satisfaction

	Non-HIV services		HIV services		All patients	
	OR	(95% CI)	OR	(95% CI)	OR	(95% CI)
Perceived quality (% very good/excellent)						
Health personnel practices and conduct	3.05***	(1.95 to 4.77)	9.49***	(2.51 to 35.93)	3.91***	(2.41 to 6.36)
Adequacy of resources and services	2.17**	(1.30 to 3.62)	3.07	(0.83 to 11.32)	2.00***	(1.38 to 2.90)
Healthcare delivery	1.61	(0.99 to 2.63)	1.04	(0.38 to 2.88)	1.57*	(1.03 to 2.39)
Accessibility of care	2.52***	(1.69 to 3.79)	1.69	(0.59 to 4.89)	1.93***	(1.35 to 2.76)
Cost of care	1.25	(0.83 to 1.89)	1.11	(0.39 to 3.16)	1.17	(0.77 to 1.77)
N	1927		398		2325	

The exponentiated coefficients shown here can be interpreted as the OR of giving a higher overall rating, for a one-unit increase in the predictor variable.

*p<0.05; **p<0.01; ***p<0.001.

In the final combined model, all of the facility, patient, visit, and perceived quality factors that were significantly associated with overall satisfaction in the first two models remained significant predictors (table 5). In addition, better self-rated health was significantly associated with higher odds of overall satisfaction for non-HIV (OR=1.29, 95% CI 1.01 to 1.66) and HIV (OR=2.68, 95% CI 1.35 to 5.30) patients. Perceived quality of healthcare delivery was an additional significant predictor for non-HIV patients (OR=1.75, 95% CI 1.13 to 2.70) and adequacy of resources and services was associated with better odds for HIV patients (OR=4.68, 95% CI 1.81 to 12.10).

**Table 5:** Ordered logistic regression results examining demographic, facility, visit, and perceived quality of care as predictors of overall patient satisfaction

	Non-HIV services		HIV services		All patients	
	OR	(95% CI)	OR	(95% CI)	OR	(95% CI)
Perceived quality (% very good/excellent)						
Health personnel practices and conduct	3.53***	(2.34 to 5.33)	11.00***	(3.97 to 30.51)	4.08***	(2.64 to 6.30)
Adequacy of resources and services	1.66*	(1.08 to 2.55)	4.68**	(1.81 to 12.10)	2.15***	(1.48 to 3.11)
Healthcare delivery	1.75*	(1.13 to 2.70)	1.02	(0.47 to 2.24)	1.68*	(1.06 to 2.67)
Accessibility of care	2.49***	(1.64 to 3.76)	1.24	(0.46 to 3.37)	2.08***	(1.45 to 2.98)
Cost of care	1.42	(0.94 to 2.16)	1.05	(0.27 to 4.15)	1.17	(0.78 to 1.77)
Facility characteristics						
MOH managed (ref: private/NGO)	0.41***	(0.27 to 0.64)	0.77	(0.45 to 1.33)	0.6	(0.29 to 1.24)
Rural (ref: urban)	0.69*	(0.48 to 0.99)	0.26***	(0.16 to 0.41)	0.47***	(0.32 to 0.69)
Hospital (ref: health centre or post)	1.78***	(1.27 to 2.48)	0.93	(0.63 to 1.39)	1.3	(0.88 to 1.93)
Patient characteristics						
Age	1	(0.99 to 1.00)	1	(0.97 to 1.02)	1	(0.99 to 1.00)
Male respondent	1.19	(0.98 to 1.45)	1.12	(0.63 to 1.99)	1.22*	(1.01 to 1.47)
Education of respondent (ref: none)						
Primary	0.82	(0.58 to 1.14)	1.33	(0.46 to 3.85)	0.89	(0.66 to 1.19)
Post-primary	0.66*	(0.46 to 0.96)	1.31	(0.42 to 4.09)	0.75	(0.53 to 1.05)
Self-rated very good or excellent health	1.29*	(1.01 to 1.66)	2.68**	(1.35 to 5.30)	1.34**	(1.11 to 1.62)
Ethnicity (ref: other)						
Tonga	0.81	(0.65 to 1.02)	0.583*	(0.37 to 0.93)	0.84	(0.69 to 1.03)
Chewa Nyanja	0.85	(0.62 to 1.16)	0.8	(0.47 to 1.35)	1.06	(0.84 to 1.33)
Patient respondent (ref: attendant)	0.89	(0.70 to 1.13)	2.21	(0.98 to 5.01)	0.93	(0.73 to 1.18)
Visit characteristics						
Travelled an hour or more	0.96	(0.77 to 1.21)	0.83	(0.48 to 1.44)	0.89	(0.72 to 1.10)
Waited an hour or more	1.12	(0.89 to 1.41)	0.68	(0.41 to 1.14)	0.96	(0.79 to 1.16)
Saw doctor or CO (ref: nurse/other)	0.86	(0.62 to 1.19)	1.3	(0.57 to 2.96)	0.9	(0.61 to 1.32)
N	1927		398		2325	

The exponentiated coefficients shown here can be interpreted as the OR of giving a higher overall rating, for a one-unit increase in the predictor variable.

*p<0.05; **p<0.01; ***p<0.001.

CO, clinical officer; MOH, Ministry of Health, Zambian government; NGO, non-governmental organisation.

## Conclusions

Providing patients with satisfactory care is an intrinsic health system goal as well as a means of driving demand for services. On average, the Zambian patients we interviewed reported overall satisfaction scores of 6.9 of 10, with substantial variation in patient opinion depending on the specific aspect of care in question and the type of facility visited.

Patients had poor perceptions of many physical characteristics, reflecting reports of weak infrastructure across Zambia and sub-Saharan Africa.^38^ ^39^ Patients were also critical of long wait times, most likely driven by human resource shortages and inefficiencies in the provision of care.^40^ ^41^ These perceptions were significantly associated with overall satisfaction in our regression analysis, suggesting that supply-side interventions to improve staffing and resource availability can also meaningfully impact patient attitudes that drive demand.^42^ ^43^

Our findings also highlight that while most patients had good perceptions of personnel, a negative interaction can have a very meaningful influence on overall satisfaction. Ratings of provider behaviour were an especially strong predictor of satisfaction for HIV patients, reinforcing qualitative research on the importance of respectfulness and confidentiality for this group.^44^

We also found that patients were less satisfied at rural, public, and lower level facilities. Previous studies have identified objective physical and human resource shortages especially affecting rural, public, low-level facilities, and we hypothesised that patients had lower satisfaction due to their perceptions of these shortages.^26^ ^27^ ^45^ However, when we controlled for these perceptions in our final model, facility management, type, and location remained significant predictors of overall satisfaction. Unless we failed to control for perceptions of a particularly important aspect of care, this could indicate that patients have inherent biases or expectations at rural, public and lower level facilities that extend beyond their actual experiences and observations. Improving satisfaction could require both objective facility improvements and efforts to alter underlying public biases. Disentangling the subjective factors underlying patient expectations and satisfaction is an important area for ongoing research.^46^

Finally, although Zambia has begun integrating the provision of HIV care with other services, HIV patients gave statistically significantly higher ratings than non-HIV patients for overall satisfaction and several technical aspects of care. Policymakers must consider the magnitude of these differences alongside their statistical significance. For instance, while the five percentage point difference in perceived drug quality was statistically significant, it may be less important than addressing the gap in drug availability (a 12 percentage point gap). Given the efficacy of ARVs, it is perhaps unsurprising that HIV patients had favourable perceptions of drug quality and treatment effectiveness.^47^ Higher ratings for equipment and drug availability could reflect concerns that HIV receives disproportionate resources and aid compared with, or even at the expense of, other services.^48–50^ However, the fact that HIV patients waited and travelled longer implies the opposite for human resources and facility density.^51^ Policymakers should carefully monitor how rapidly scaling up HIV services has impacted the experiences and satisfaction of HIV and non-HIV patients, and consider how HIV supply chain successes could be extended to other resources.^15^ ^52^

Our findings must be interpreted in the light of the following limitations. First, exit interviews, by nature, only include patients who sought care. Therefore, our findings only reflect the opinions of patients who interacted with the health system, not those of the general population. For instance, our sample of patients may have higher satisfaction than the overall population, because individuals who are satisfied with the health services available are more likely to seek care than those who had a bad experience.^5^ Second, our systematic sample may not truly represent the patient population, as we did not randomly sample patients from a roster. Also, the sample was not specifically designed to target HIV patients, limiting the size and representativeness of this subsample. Third, while exit interviews offer the advantage of reduced recall bias, conducting interviews at the facility may make patients hesitant to express their true opinions. Fourth, our findings should not be interpreted as a causal association between perceived quality of care and patient satisfaction. A more nuanced econometric model such as structural econometric modelling or path analysis may be better suited to establish the structures of causality. It is likely that some observed and unobserved facility and patient characteristics may affect different dimensions of perceived quality of care and overall satisfaction. Furthermore, these characteristics may affect overall satisfaction indirectly through the quality of care ratings. Our analyses do not address these concerns, and therefore results should be interpreted with caution. Finally, patient perceptions and satisfaction may have changed since the survey was conducted in 2012. Ideally, Zambia should implement an ongoing system to monitor levels and trends in patient satisfaction, as many developed countries have.^3^ To the best of our knowledge, our survey is currently the most recent national satisfaction survey of its scale, and the results raise substantial equity concerns that should be closely evaluated today and addressed. It provides the timeliest information available on Zambian patient satisfaction and can serve as a baseline measurement for tracking and evaluation purposes.

Patient satisfaction is an important goal for both intrinsic patient rights and health outcomes. From a rights perspective, systematic variation in satisfaction depending on facility location, management and level raises concerns about the equity of care in Zambia. Additionally, previous literature has demonstrated that low satisfaction is an important driver of two key challenges facing the Zambian health system: healthcare-seeking behaviour and adherence to treatment, particularly for HIV. Our findings provide a road map to policymakers as to which aspects of care should be prioritised to have the greatest impact on overall satisfaction, including training and incentivising staff to treat all patients with dignity and improving resource availability. The results implore policymakers to prioritise satisfaction interventions at rural, public, and lower level facilities, keeping in mind that perceptions of these facilities may be driven by both tangible factors and internalised beliefs or biases.
